# ITPR1 Mutation Contributes to Hemifacial Microsomia Spectrum

**DOI:** 10.3389/fgene.2021.616329

**Published:** 2021-03-04

**Authors:** Zhixu Liu, Hao Sun, Jiewen Dai, Xiaochen Xue, Jian Sun, Xudong Wang

**Affiliations:** ^1^Department of Oral and Cranio-Maxillofacial Surgery, Shanghai Ninth People’s Hospital, Shanghai Jiao Tong University School of Medicine, Shanghai, China; ^2^Key Laboratory of Spine and Spinal Cord Injury Repair and Regeneration, Ministry of Education, Tongji University, Shanghai, China; ^3^National Clinical Research Center for Oral Diseases, Shanghai Key Laboratory of Stomatology, Shanghai Research Institute of Stomatology, Shanghai, China

**Keywords:** hemifacial microsomia, ITPR1, PLCB4, DLX5, DLX6, zebrafish

## Abstract

Hemifacial microsomia (HM) is a craniofacial congenital defect involving the first and second branchial arch, mainly characterized by ocular, ear, maxilla-zygoma complex, mandible, and facial nerve malformation. HM follows autosomal dominant inheritance. Whole-exome sequencing of a family revealed a missense mutation in a highly conserved domain of *ITPR1*. ITPR1 is a calcium ion channel. By studying *ITPR1*’s expression pattern, we found that ITPR1 participated in craniofacial development, especially the organs that corresponded to the phenotype of HM. In zebrafish, *itpr1b*, which is homologous to human *ITPR1*, is closely related to craniofacial bone formation. The knocking down of *itpr1b* in zebrafish could lead to a remarkable decrease in craniofacial skeleton formation. qRT-PCR suggested that knockdown of *itpr1b* could increase the expression of *plcb4* while decreasing the mRNA level of Dlx5/6. Our findings highlighted ITPR1’s role in craniofacial formation for the first time and suggested that *ITPR1* mutation contributes to human HM.

## Introduction

Hemifacial microsomia (HM, OMIM:164210) is the second most common craniofacial birth defect after cleft lip and palate. The estimated incidence of HM is about 1/3500 to 1/5600 among live births (Tuin et al., 2015; Brandstetter and Patel, 2016). HM affects the development of the first and second branchial arch derivatives, resulting in defects in the orbits, eyelids, mandible, maxilla-zygoma complex, external ear, middle ear, facial nerves, masticatory, and facial muscles. In some cases, the parotid gland, vertebral system, heart, kidney, etc. may also be affected (Tuin et al., 2015; Brandstetter and Patel, 2016).

Although the genetic knowledge of human diseases has been greatly expanded in the past decades, the etiology of HM remains elusive. Both genetic and environmental factors have been suggested as possible causes of HM (Beleza-Meireles et al., 2014). Maternal diabetes during pregnancy, smoking, twinning, and vasoactive drugs are considered as the environmental causes of some HM cases (Beleza-Meireles et al., 2014). While most HM cases are sporadic, some familial cases have been reported, suggesting genetic mutations as contributing factors for HM. Thus, it has been suggested that the mutations in specific genes could be the major causal factors of HM, with environmental factors further promoting or magnifying the genes’ effects. However, as only 2% of HM patients with family history has been documented, it has been challenging to dissect the genetic causes of HM (Brandstetter and Patel, 2016). After analyzing 74 probands, Kaye et al. (1992) concluded that HM followed autosomal dominant inheritance. Most of the studies considered the autosomal dominant or autosomal recessive inheritance with various chromosomal mutations and genomic imbalances are the causes of HM. From a cohort of 169 patients, Estelle Lopez et al. found MYT1 mutation in a sporadic case by whole exon sequencing and detected one heterozygous missense mutation in another patient (Lopez et al., 2016). Other genes related to HM included SALL1, BAPX1, TCOF1, and EFTUD2 (Fischer et al., 2006; Kosaki et al., 2007; Su et al., 2012; Dai et al., 2016; Rengasamy Venugopalan et al., 2017). Here, we present the genetic studies of a family with autosomal dominant HM and demonstrate the role of the EDN-PLC-DLX5/6 regulatory cascade in HM.

## Materials and Methods

### Patients

Participants enrolled in this study were approved by the Institutional Review Board (IRB) at Shanghai Ninth People’s Hospital, Shanghai Jiao Tong University School of Medicine, the ethics committee of the clinical study. Informed consent for blood samples and DNA storage and genetic analysis was obtained from all subjects. All procedures in this study involving human participants were performed following the ethical standards of the institutional and/or national research committee and with the 1964 Helsinki declaration and its later amendments or comparable ethical standards.

### Whole-Exome Sequencing Analysis

The qualified genomic DNA sample was randomly fragmented. Library construction was performed on double-stranded DNA, and the size of library fragments was mainly distributed between 200 bp to 300 bp. The extracted DNA was amplified by ligation-mediated PCR, purified, and hybridized (captured) to the target regions. The captured PCR products were then subjected to Agilent 2100 Bioanalyzer and quantitative PCR to estimate the magnitude of enrichment. High-throughput sequencing was completed on Illumina Hiseq Platforms to ensured that each sample met the desired sequencing coverage (90X). Pair-end reads were generated by Illumina Base-calling Software and stored in the FASTQ format. The data of each sample were mapped to the human reference genome (GRCh37/hg19). The alignment was processed by a Burrows-Wheeler Aligner (BWA). Variant sites were called with the Genome Analysis Toolkit (GATK^1^). Trio WES was performed for the proband and his parents with a mean depth of coverage for each sample of 90-fold, 97.48% of the targeted bases had at least 10-fold coverage or greater. The analysis was completed using custom-developed software (Children’s Hospital of Fudan University, Shanghai Key Laboratory of Birth Defects).

### Sanger Sequencing

Sanger sequencing was performed by standard methods on the mutation portion of *ITPR1*, and was amplified with primers 5′-CGTTTTGAGTTTGAAGGCGTTT-3′ and 5′-CATCTTGCGCCAATTCCCG-3′ (designed with https://www.ncbi.nlm.nih.gov/tools/primer-blast/index.cgi?LINK_LOC=BlastHome). PCR products were amplified using 50 ng of DNA template and standard PCR reagents (Takara, Japan) on an ABI Verti Thermocycler (Applied Biosystems, United States). PCR products were sent for Sanger sequencing (Sangon Biotech Co., Ltd., China).

### Molecular Modeling of the ITPR1 Protein

We used a three-dimensional computer model and the crystal structure of mouse ITPR1 as a template (PDB ID: 5 × 9z) to predict the possible impact of an amino acid substitution on the structure and function of the ITPR1 protein. The human ITPR1 was modeled by SWISS-MODEL at a resolution of 7.31Å.

### Mouse Immunofluorescence Staining

All applicable institutional and/or national guidelines for the care and use of animals were followed. Wild-type mouse embryos were collected and processed for frozen sections as previously reported (sections were air-dried for 10 min at room temperature, and stored at −20°C) (Hisatsune et al., 2013). Immunofluorescent staining was performed using frozen sections following standard protocols. Antibodies used included rabbit anti-ITPR1 (1:250, IP3 receptor 1 polyclonal antibody, Invitrogen, United States) (Gennarino et al., 2015; Garland et al., 2017) and goat anti-Rb IgG (1:1,000, Alexa Flour 488, Invitrogen, United States).

### Zebrafish Morpholino Injections

#### Zebrafish Maintenance and Morpholino Injections

Adult wild-type zebrafish were maintained at 28.5°C in a 14 h light/10 h dark cycle. Five to six pairs of zebrafish were set up for natural mating. On average, 200–300 embryos were generated. Embryos were maintained at 28.5°C in fish water (0.2% Instant Ocean Salt in deionized water). The zebrafish facility at Shanghai Research Center for Model Organisms is accredited by the Association for Assessment and Accreditation of Laboratory Animal Care (AAALAC) International.

Morpholino (MO) was designed using GeneTools^2^. The sequences of the *itpr1b* translation-blocking and splice-blocking MOs were 5′-CATCTTGTCCGACATTTTGCTCCAC-3′ (ATG-MO) and 5′-TGACAAAGCACAAGTGAACTCACGT-3′ (E4I4-MO), respectively (Supplementary Figure 1A). The sequence for the standard control MO was 5′-CCTCTTACCTCAGTTACAATTTATA-3′ (Gene Tools). Antisense MOs were microinjected into fertilized one-cell to-two cell stage embryos according to standard protocols. The amount of the MOs used for injection is as follows: Control-MO and e4i4-MO, 4 ng per embryo; ATG-MO, 4 ng per embryo. The zebrafish were collected at 120 h post-fertilization (hpf). The effectiveness of *itpr1b* knockdown (*itpr1b*-e4i4-MO) was confirmed by RT-qPCR at 120 hpf (Supplementary Figure 1B).

### Calcein Staining for Zebrafish

At the end of treatment, 120 hpf zebrafish were washed with fish water three times and immersed in a 0.2% calcein solution for 10 min. Next, zebrafish were rinsed thoroughly in fish water three times (5 min/wash) and anesthetized with 0.016% MS-222 (tricaine methanesulfonate, Sigma-Aldrich, Germany). Zebrafish were then oriented on the ventral side and mounted with 3% methylcellulose (Sigma-Aldrich, Germany) in a depression slide for observation by fluorescence microscopy (Du et al., 2001; Hosen et al., 2013). Larvae were analyzed with a Nikon SMZ 1500 Fluorescence microscope and subsequently photographed with digital cameras. The relative fluorescence intensity (RFI) of head skeleton bone mass per animal was quantified using morphometric analysis (NIS-Elements D3.1, Japan). To optimally visualize the expression patterns, a subset of images was adjusted for brightness, contrast, hue, and saturation with Adobe Photoshop 7.0 software (Adobe, United States). Ten animals for each treatment were quantified and the total signal per animal was averaged.

### Quantitative Real-Time PCR for Zebrafish

Total RNA was extracted from 30 to 50 embryos per group in Trizol (Roche, United States) according to the manufacturer’s instructions. RNA was reverse transcribed using the PrimeScript RT reagent Kit with gDNA Eraser (Takara, Japan). Quantification of *itpr1b* expression was performed in triplicates using the Bio-rad iQ SYBR Green Supermix (Bio-rad, United States) with detection on the Realplex system (Eppendorf, United States). Relative gene expression quantification was performed based on the comparative threshold cycle method (2^–ΔΔCt^) using *ef1*α as an endogenous control gene. Primer sequences of *itpr1b*: forward-GTAAG CTGCTGGGAACGGTGAT and reverse-TGTAGAAGGGC TGGATGTAAA. Primer sequences of *ef1*α (internal control): forward-GGAAATTCGAGACCAGCAAATAC, reverse-GATAC CAGCCTCAAACTCACC. Primer sequences for *plcb4* in qRT PCR: forward-TCCGATGACGTGCCTGAAAA, and reverse-GAAGAGCTCCTCGATGTCGG. Primer sequences of *dlx5*: forward-ACTATGGATATGTGACTCAAGGC and reverse-TGTGACTTGTGAACGGTGCT. Primer sequences of *dlx6*: forward-ACCGTTTCCAGCAGACTCAAT and reverse-ACCGTTTCCAGCAGACTCAAT.

## Results

### Manifestations of Proband and His Father With HM

**FIGURE 1:**
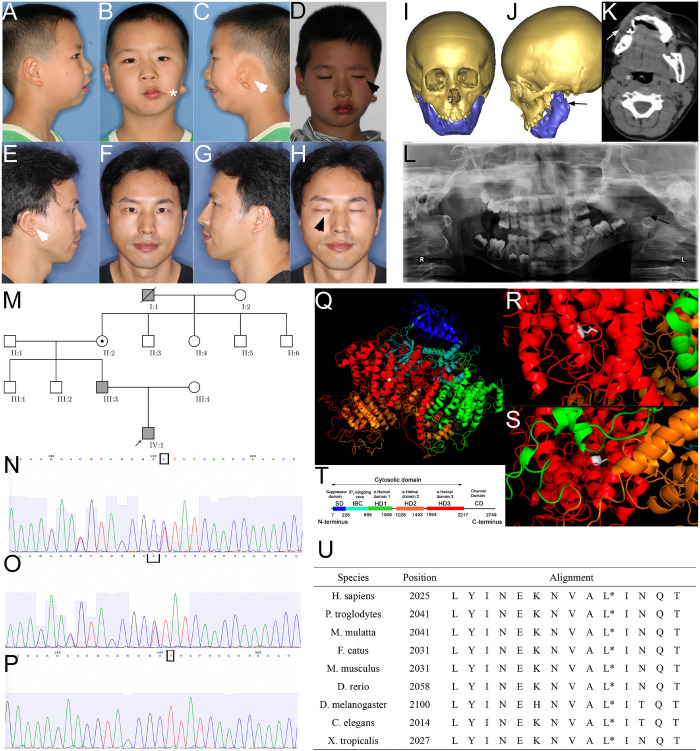
Phenotype and pedigrees of the proband with ITPR1 mutation. **(A–H)** Photos of the proband and his father, note the facial asymmetry, microtia (white arrowhead), dysfunction of the zygomatic branch of the facial nerve (black arrowhead), proband also suffered from macrostomia (star). **(I–L)** Facial CT scans with bone reconstruction and panoramic film of the proband showed hypoplasia of the left mandible ramus (black arrow) and the soft tissues (white arrow). **(M)** Pedigrees of the family, the proband, proband’s father, the proband’s great grandfather diagnosed with HM. **(N–P)** Sanger sequencing verified the mutation in the proband **(N)**, his father **(O)**, and proband’s mother. **(Q–T)** Molecular modeling by PDB’s information, blue indicates the suppressor domain, cyan represented IP3 binding core, green, orange, and red represented α-helical domain 1, 2, and 3, respectively. White indicates the point of a missense mutation, **(R)** leucine of the wild-type, which is a short-chain amino acid, **(S)** to proline (mutant), which has a benzene ring. **(T)** Domain illustration of ITPR1. **(U)** Alignment of amino acids, spanning 14 residues in diverse species, showing that this position is highly conserved (highlighted with red circle).

The proband was a 6-year-old male at the time of enrollment in the study. The major clinical phenotypes included gross facial asymmetry, left ear microtia, absence of left ear canal and conductive hearing loss, dysfunction of the zygomatic branch of the left facial nerve, and macrostomia on the left side (Figures 1A–D). Because the osteogenesis distraction procedure was performed on the affected side of the proband to correct his facial asymmetry, a craniofacial computed tomography (CT) scan was acquired. CT images documented the absence of the left parotid gland and hypoplastic left masseter (Figure 1K). Consistent with his ear deformity, a CT scan also indicated stenosis of the left auditory canal (Figure 1J). A reconstructed craniofacial 3D model revealed his left orbit was smaller and superior dislocated (Figures 1I,J). The left temporomandibular joint was dysmorphic, the residual condyle was flat, the coronal process was missing, and the mandibular ramus was shorter (Figures 1I–L).

Proband’s father had a minor but similar phenotype to the proband (son): slightly facial asymmetry, smaller mandible on the right side, right auricular deformity (post-ear-reconstructed surgery), right congenital aural atresia, dysfunction of the zygomatic branch of the right facial nerve (Figures 1E–H). The father has two healthy siblings. The father recalled his grandfather on his mother’s side, who had passed away, shared a similar phenotype that includes auricular deformity on the left side, facial asymmetry, though the degree was unknown. The father’s mother and her four siblings are healthy without observable facial deformity (Figure 1M).

### Genetic Analysis Indicated ITPR1 Mutation Could Be the Cause of HM

To investigate the genetic underpinnings for the HM phenotype in the proband and his father, we performed whole-exome sequencing (WES) analysis on the proband and his parents. Sequence analysis revealed a total of 508 variants in the proband, among which 125 were un-recorded in the normal human gene bank (1000 genes, ExAC). In those 125 variants, only five variants were shared by both the father and the son (Table 1). Further analysis suggested that only the variants associated with two genes, inositol 1,4,5-triphosphate receptor, type 1 (ITPR1:NM_ 001099952:c.5975T > C:p.Leu1992Pro, ITPR1:NM_001168272: c.6074T > C:p.Leu2025Pro, ITPR1:XM_005265108:c.6122T > C:p.Leu2041Pro), and Rap guanine nucleotide exchange factor (GEF) 2 (RAPGEF2, NM_014247:exon23: c.4218_4218delinsCA) were considered to be pathogenic (Table 2). The RAPGEF2 mutation detected by WES was a frameshift mutation. And the non-synonymous mutation in ITPR1 detected by WES was predicted to be detrimental by both SIFT^3^ (Sim et al., 2012) and polyphen2^4^. We subsequently performed Sanger sequencing of ITPR1 and RAPGEF2 in all three family members to validate these potentially pathogenic mutations. However, only the heterozygous variant in ITPR1 was verified (Figures 1N–P). Therefore, we reasoned that the ITPR1 mutation was the most probable candidate underlying the HM phenotype in the proband. To further understand the functional impact of the mutation, we performed molecular modeling of the ITPR1 structure. Molecular modeling of ITPR1 illustrated that the mutation is located in the HD3, with a proline replacing the original leucine residue, potentially affecting the function of the protein (Figures 1Q–T).

**TABLE 1:** Filtering strategy for trio exome.

Filter	Variants
Total variants	508 variants
Coding, non-synonymous	439 variants
Un-recorded in normal human gene bank (1,000 genes, ExAC^‡^)	125 variants
Inheritance (only father and child involved)	5 variants (ITPR1, RAPGEF2, CEP170, HNRNPU, PEAR1)
Protein damage prediction	ITPR1, RAPGEF2
Sanger sequencing verification	ITPR1

*^‡^ExAC, exome aggregation consortium.*

**TABLE 2:** *In silico* predictions for variant.

Approved name	Position	Mutation	Mutation type	Depth	SIFT score	SIFT prediction	Polyphen2 score	Polyphen2 prediction
ITPR1	chr3:4817065	T to C	Non-synonymous	0/1:110,132:242	0	D	1	D
RAPGEF2	chr4:160277054	C to CA	frameshift Substitution	0/1:38,5:43	–	–	–	–
CEP170	chr1:243328779	T to C	Non-synonymous	0/1:37,44:81	0.3	T	0.298	B
HNRNPU	chr1:245017780	C to T	Non-synonymous	0/1:32,34:66	0.83	T	0.048	B
PEAR1	chr1:156874628	A to G	Non-synonymous	0/1:27,22:49	0.16	T	0.001	B

### *Itpr1* Expression in Craniofacial Region

**FIGURE 2:**
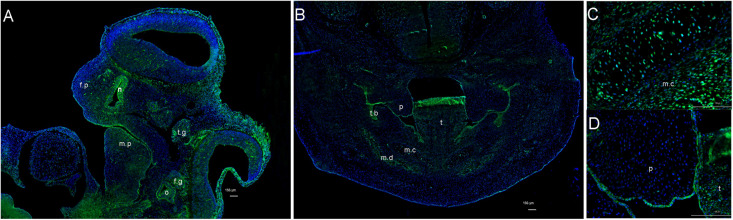
Expression pattern of *ITPR1*. **(A)** At E 11.5, *ITPR1* is expressed in the frontonasal process, first branchial arch, and second branchial arch, like the mandibular process, nasal pit, otocyst, trigeminal (V) ganglia, and facial-acoustic (VII-VIII) ganglia. **(B)** By E13.5, *ITPR1* shows significant expression at the craniofacial region, like the tooth bud, mandibular mesenchyme, **(C)** epithelial layer of palate shelves, and **(D)** Meckel’s cartilage. ITPR1 is marked in green fluorescence. DNA was stained by DAPI and is marked in blue fluorescence. Abbreviations are as follows: f.p, frontonasal process; f.n, nasal pit; m.p, mandibular process; o, otocyst; t.g, trigeminal (V) ganglia; f.g, facioacoustic (VII-VIII) ganglia; p, palate, t, tongue; t.b, tooth bud; m.c, Meckel’s cartilage; m.d, mandible.

To further understand ITPR1’s relationship with HM, we investigated the expression pattern of *Itpr1* by performing immunofluorescence in mouse embryos. At E11.5, right before the maxilla and mandible begin to merge, we could detect the expression of ITPR1 in both the first and second branchial arches, including the mandibular and maxillary processes, nasal pits, otocyst, and facial-acoustic (VII-VIII) ganglia (Figure 2A). By E13.5, ITPR1 was strongly expressed at the face region, including the teeth bud, the epithelial layer of palate shelves, mandible, and Meckel’s cartilage (Figures 2B–D). These results revealed ITPR1’s role in craniomaxillofacial and accord with the phenotypes of the proband and his father.

### Functional Analysis of the *ITPR1* Variants in Zebrafish

**FIGURE 3:**
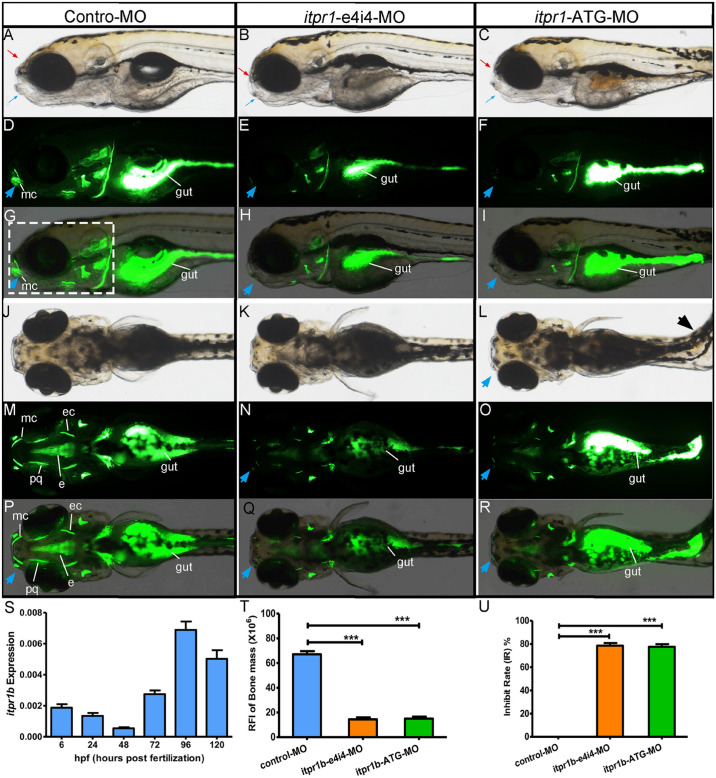
Gross morphology of zebrafish with *itpr1b* knocked down at 5-dpf. Lateral view of control MO-injected zebrafish embryos and embryos injected with *itpr1b* morpholino oligonucleotides (MO) **(A–I)**. Calcium staining by calcein in MO-control, MO-itpr1-e4i4, and MO-itpr1-ATG morphants at 120 hpf (hours post-fertilization). Both MO-itpr1-e4i4, MO-itpr1-ATG morphants showed bimaxillary retrognathia [**(A–C)**, red arrowed indicated the upper jaw, blue arrowed indicated the lower jaw]. The gut is filled with calcein **(D–I,M–R)**. Fluorescent signals were apparent in 120-hpf embryos and restricted to the head skeleton **(D–I,M–R)**. For MO-itpr1-e4i4 and MO-itpr1-e4i4 morphants, Meckel’s cartilage can barely be recognized [blue arrowhead, **(E,F,H,I)**]. From the ventral view **(J–R)**, scoliosis can be observed in MO-itpr1-ATG (black arrowhead), Meckel’s cartilage is undistinguished (blue arrowhead). Fluorescent signals were greatly reduced in palatoquadrate and ethmoid and ectopterygoid. **(S)** *itpr1b* expression was induced in 72-hpf embryos, further increased at 96 hpf, and remained elevated at 120 hpf. **(T)** Graph presenting the quantification of the relative fluorescence intensity (RFI) of head skeleton bone mass (*N* = 10, ANOVA, ***P < 0.001). **(U)** Graph presenting the quantification of the inhibit rate (IR) of head skeleton bone mass (*N* = 10, ANOVA, ***P < 0.001). The region used to calculate bone mass is shown in panel **(G)** highlighted with the dashed rectangle. mc, Meckel’s cartilage; pq, palatoquadrate; ec, ectopterygoid; e, ethmoid.

Zebrafish is a powerful animal model for screening and validating gene mutations (Adamson et al., 2018). The zebrafish homolog of the human *ITPR1* gene, *itpr1b*, exhibits high sequence identity (87.4% of the protein sequence and 76.1% of the DNA sequences) with its human counterpart. *Itpr1b* expression was monitored by qRT-PCR at six embryo developmental stages (6 hpf, 24 hpf, 48 hpf, 72 hpf, 96 hpf, 120 hpf, hpf is short for hours post-fertilization). We found that the transcription of *itpr1b* started to be induced at 72 hpf, significantly increased at 96 hpf, and remained elevated at 120 hpf (Figure 3S). We further examined the temporal pattern of the head skeleton formation in zebrafish. No calcification signals could be detected in zebrafish embryos up to 96 hpf. At 120 hpf, calcification signals became apparent and restricted to the head (Figures 3D,G,M,P). These findings suggest that *itpr1b* may play an important role in craniofacial bone calcification, as most of the head skeleton begins to form from 96 hpf to 120 hpf (Du et al., 2001; Hosen et al., 2013).

**FIGURE 4:**
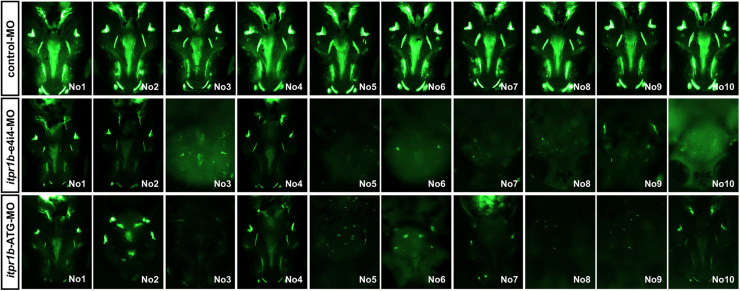
Zebrafish have been picked up to take photos and calculate the craniofacial skeleton mass.

Transient knockdown of *itpr1b* with either MO-*iptr1b*-ATG (translation blocking) or MO-*itpr1b*-e4i4 (splice blocking) at 120 hpf (Supplementary Figure 1A) resulted in severe defects in head skeleton morphology. Compared with control MO, zebrafish injected with MO-*itpr1b*-ATG and MO-*itpr1b*-e4i4 exhibited retrognathia (sizes of both upper and lower jaw were reduced), vertebral defects such as scoliosis (Figures 3A–C,J–L), with the ratios of skeletal deformation elevated to 61.54% and 31.46%, respectively (Table 3). Meckel’s cartilage, palatoquadrate, ethmoid, and ectopterygoid were easy to distinguish in the control by calcein staining, while in the MO-*iptr1b-*ATG and MO-*itpr1b*-e4i4, the fluorescent signals could be hardly detected (Figures 3D–I,M–R). Scoliosis can also be observed in MO-*iptr1b*-ATG and MO-*itpr1b-*e4i4 groups, with lower penetrance compared with craniofacial deformities (Figure 3L). We randomly selected 10 zebrafish from each group (for MO-*iptr1b*-ATG and MO-*itpr1b*-e4i4 groups, only those showing defects were chosen) for quantification evaluation after imaging (Figure 4). Quantification of the relative fluorescence intensity (RFI) of head skeletal bone indicated a significantly decreased skeleton bone mass in the mutant groups (Figures 3T,U).

**TABLE 3:** Embryos with defects in zebrafish.

Group	Total number	Embryos with defects (number)	Embryos with defects (%)
Control MO-5dpf	98	0	0
MO-e4i4-5dpf	89	28	31.46
MO-ATG-5dpf	78	48	61.54

We further demonstrated that ITPR1 regulates skeletal development *via* modulation of the EDN-PLC-DLX5/6 regulatory axis. EDN1 (endothelin 1, MIM 131240) and its G protein-coupled receptor EDNRA (endothelin receptor A, MIM 131243) play important roles in mandibular development (Tavares et al., 2012; Kim et al., 2013; Gordon et al., 2015). EDNRA activates PLCB4 (phospholipase C beta 4), which cleaves the PIP2 and triggers the PLC signaling cascade (Marinissen and Gutkind, 2001), leading to the expression of key transcriptional regulators of skeletal development, such as *Dlx5* and *Dlx6*. In zebrafish, mutations of *edn1* could lead to a reduction in lower jaw size (Miller et al., 2000; Kimmel et al., 2003). The deletion of *Edn1* or *Ednra1* in mice can result in severe mandibular deformity, as mandibular arch-derived structures transform into maxillary-like structures (Sato et al., 2008; Tavares et al., 2012). PLCB4, EDN, and EDNRA are closely related to human auriculocondylar syndrome (ACS, MIM #614669, #602483, #615706), which shares similar phenotypes with OAVS like ear and mandibular deformities (Rieder et al., 2012; Romanelli Tavares et al., 2017), suggesting a functional link with ITPR1.

**FIGURE 5:**
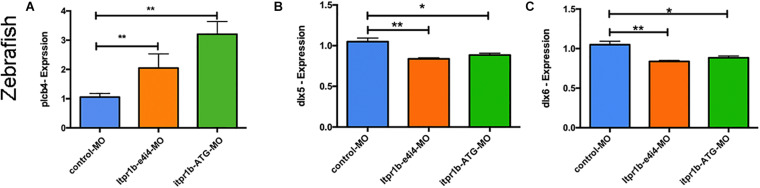
ITPR1 involved in the EDN-PLC-DLX5/6 axis. **(A)** qRT-PCR analysis of *plcb4* expression in zebrafish with *itpr1b* knockdown was shown, *plcb4 transcripts* increased in both MO-itpr1-e4i4 and MO-itpr1-ATG morphants. The relative transcript level was calculated as fold change using the 2^– ΔΔ^Ct method (ANOVA) (*N* = 5). **(B,C)** *dlx5* and *dlx6 transcripts* decreased in both MO-itpr1-e4i4 and MO-itpr1-ATG morphants. (ANOVA, *N* = 5).

The mRNA level of *plcb4* increased in the MO-*iptr1b*-ATG and MO-*itpr1b*-e4i4-treated zebrafish compared to the control (Figure 5A), which may indicate a compensatory effect in response to the ITPR1 deficiency. Since ITPR1 is a calcium gate in the PLC (phospholipase C) signaling pathway, we examined whether the knockdown of ITPR1 affects PLC signaling and the expression of DLX5/DLX6. By performing qRT-PCR, we confirmed that the transcripts levels of *dlx5* and *dlx6* were reduced in zebrafish treated with MO-*iptr1b*-ATG and MO-*itpr1b*-e4i4 (Figures 5B,C). Thus, ITPR1 may profoundly influence the activity of the EDN-EDNRA-DLX5/DLX6 pathway.

## Discussion

In the present study, we identified a missense mutation in the *ITPR1* gene in a family with HM. ITPR1 is an intracellular IP_3_ (inositol 1,4,5 triphosphate)-gated calcium ions channel. In fish, amphibians, and mammals, three paralogs of ITPR can be identified (ITPR1, ITPR2, ITPR3), among which *ITPR1* is the most widely expressed paralog (Gerber et al., 2016). Although the expression pattern of various ITPR subtypes overlap, the heterogeneity in sequences suggests that ITPRs are functionally diverse (Smutzer et al., 1997). ITPR1 releases Ca^2+^ from the endoplasmic reticulum into the cytosol by responding to IP_3_, which is cleaved from phosphatidylinositol (PIP2) in a G protein-dependent manner (Marinissen and Gutkind, 2001; Decuypere et al., 2015). ITPR1 is usually composed of six domains, which include an N-terminal suppression domain (SD), IP_3_ –binding core (IBC), three curvature α-helical domains 1–3 (HD1-3), and a calcium channel domain (Figure 1T; Hamada et al., 2017). Among all the HD domains, the HD3 domain has been suggested to be the most essential for ITPR1 function, as it connects to the calcium channel domain (Hamada et al., 2017). The mutation in *ITPR1* of the proband and his father reported in this study was located at a position in the HD3 domain that is highly conserved among the nine species we had studied (Figure 1U), strongly suggesting that the mutation may lead to ITPR1 dysfunction during human embryo development.

ITPR1 has been widely studied in the nervous system, and linked to human spinocerebellar ataxia 15 (MIM 606658), Gillespie syndrome (MIM 206700), and spinocerebellar ataxia 29 (MIM 117360) (van de Leemput et al., 2007; Das et al., 2017). However, the role of ITPR1 in craniofacial bone development has not been explicitly studied. A previous study showed that the expression of ITPR1 can be detected as early as 5.5 pdc in mouse embryos (Rosemblit et al., 1999). ITPR1 is expressed in first and second branchial arches during embryonic development, especially in the mandibular process, otocyst, and facial-acoustic (VII-VIII) ganglia. The phenotypes of the proband and his father are restricted in the locations where *ITPR1* expresses during embryonic development, suggesting that ITPR1 may be functionally involved in craniofacial development.

We further assessed the function of ITPR1 in craniofacial skeletal development *via* genetic manipulation in zebrafish. The expression of *itpr1b*, a zebrafish homolog to human *ITPR1*, can be detected at 6 hpf. *itpr1b* expression was the highest from 96 hpf to 120 hpf, a period important for calcium deposition during craniofacial skeleton bone formation (Hosen et al., 2013). Two MOs were designed to downregulate *itpr1b* expression in zebrafish, with one targeting ATG (MO-itpr1b-ATG) to block ribosome from assembling and the other modified mRNA translation by targeting exon 4 (MO-itpr1b-e4i4). We found the skeleton mass of the head decreased in both MO-itpr1b-ATG and MO-itpr1b-e4i4, especially in structures related to the Meckel’s cartilage and the palatoquadrate. Some of the zebrafish also suffered from scoliosis, further indicating craniofacial skeleton deformation. These results revealed the important role of ITPR1 in craniofacial bone formation, especially calcium deposition. Consistently with these findings, spine deformities are also common in HM patients (Caron et al., 2017).

**FIGURE 6:**
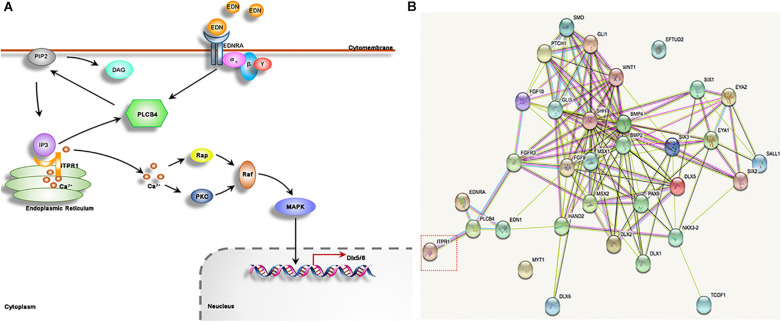
ITPR1 is involved in different pathways. **(A)** It has been illustrated by several reports that the EDN-EDNRA-G protein α_q_-DLX5/6 axis has important roles in mandible formation. EDN binds to receptor EDNRA, a G-protein that can activate PLCB4. PLCB4 splits PIP2, membrane-bound phospholipids, into DAG and IP3. IP3 binds to its receptor ITPR1, a calcium release channel, to allow the passage of calcium to move through. Calcium works to activate PKC and Rap, and stimulate MAPK signaling, which can regulate Dlx5 and Dlx6 expression. **(B)** The network of protein-protein interaction is shown, produced by STRING software (https://string-db.org), including ITPR1 and its closest partner PLCB4. EDN1 and EDNRA genes may be involved in craniofacial development especially those reported as a causative protein of human mandibular deformities, and among those with gene mutation have been reported in HM patients, like MYT1 and EFTUD2.

We further show that ITPR1 is involved in the EDN-PLC-DLX5/6 regulatory axis. PLCB4 is a core molecule in G protein-PLC signaling. After being activated by cell surface receptors (e.g., EDNRA), PLCB4 will cleave the PIP2 and regulate the opening of the ITPR1-calcium channel (Marinissen and Gutkind, 2001), eventually leading to transcription activation of Dlx5/6 (Rieder et al., 2012; Figure 6A). *Plcb4*^–/–^ mutant mice exhibited cerebellar hypoplasia and ataxia (Miyata et al., 2001), closely mirroring neuronal phenotypes associated with IPTR1 mutations, indicating a functional connection between IPTR1 and PLCB1. The EDN-PLC-DLX5/6 regulatory axis plays an important role in craniofacial development. Each targeted mutation of *Edn1*, *Ednra*, and G protein alpha subunits (Gα_q_) in mice led to a reduction in jaw size (Ivey et al., 2003; Yanagisawa et al., 2003; Ozeki et al., 2004; Vieux-Rochas et al., 2010). Notably, *EDN1* and PLCB4 mutations are linked to human ACS (Rieder et al., 2012; Gordon et al., 2013; Romanelli Tavares et al., 2017), which exhibits a typical phenotype including micrognathia, small mandibular condyle, and auricular malformation with a question mark earlobe (Gordon et al., 2013; Kido et al., 2013). The phenotypes of ACS are commonly observed in HM patients, suggesting the defects in EDN-PLC-DLX5/6 signaling may underlie the craniofacial defects in HM. Here we show that the downregulation of *itpr1b* in zebrafish led to the upregulation of *plcb4*, while downregulating *Dlx5 and Dlx6*. Taken together, we suggest that ITPR1 is involved in the EDN-PLC-DLX5/6 axis. The increase of PLCB4 expression may stimulate the capacity of ITPRs to release the calcium from the endoplasmic reticulum, thus serving as a compensatory mechanism to the deficiency of ITPR1.

Craniofacial development is a highly sophisticated process. A lot of genes and pathways, such as DLXs, FGFs, WNT, EDN, HH, and SIXs, have been shown to play important roles in craniofacial development (Figure 6B; Minoux and Rijli, 2010; Garcez et al., 2014). HM exhibits a wide spectrum of phenotypes involving different organs, in which the phenotypes of each organ can range from minor to severe in patients. A large number of chromosomal anomalies have been identified in patients with HM. However, the main genetic causes for HM remain obscure. We speculate that the phenotypic variability of HM may stem from the inherent redundancy and complex genetic interactions among the genes involved in craniofacial development. Though mutations in genes could lead to hereditary susceptibility of HM. In most cases, the heredity deformation could be rescued by homolog genes and compensatory genetic by-paths. In this study, knockdown of *itpr1b* in zebrafish resulted in highly variable jaw deformation ratios ranging from 31.46% to 61.45%. Though these variations may be partially caused by variations in experimental manipulation, it could also indicate the complex genetic interactions in EDN-PLC-DLX5/6 signaling. Consistent with this notion, *Plcb4* mutation in mice can cause ataxia, but human patients, diagnosed with ACS, showed no sign of ataxia. Furthermore, *ITPR1* mutation in humans can cause Gillespie syndrome or ataxia, while in our HM family no ataxia can be observed, suggesting that ITPR1’s function in the central neural system may be complemented by other ITPR family members or pathways. Therefore, interruption of EDN-PLC-DLX5/6 signaling can be partly, or even totally, rescued in some cases through compensatory mechanisms, resulting in a high degree of phenotypic and pathological variability.

We note that only a small number of patients are involved in this study. However, as the causative of HM is still under dispute, we think our study is still valuable in that it provides a novel candidate gene - ITPR1 and demonstrated its function in skeletal development in zebrafish. Future investigations in mouse models defective in IPTR1 will further reveal the roles of ITPR1 in craniofacial development and the pathological manifestation of HM.

## Conclusion

We reported the identification of a mutation occurring at a conserved location in *the ITPR1* gene in a HM family. Dysfunction of ITPR1 can affect the branchial arch development and contribute to craniofacial deformities. ITPR1 is involved in the regulation of the EDN-PLC-DLX5/6 signaling axis.

## Data Availability Statement

The datasets presented in this study can be found in online repositories. The names of the repository/repositories and accession number(s) can be found below: https://www.ncbi.nlm.nih.gov/sra/PRJNA681258.

## Ethics Statement

The studies involving human participants were reviewed and approved by Institutional Review Board (IRB) at Shanghai Ninth People’s Hospital. Written informed consent to participate in this study was provided by the participants’ legal guardian/next of kin. The animal study was reviewed and approved by Institutional Review Board (IRB) at Shanghai Ninth People’s Hospital. Written informed consent was obtained from the individual(s), and minor(s)’ legal guardian/next of kin, for the publication of any potentially identifiable images or data included in this article.

## Author Contributions

XW designed the study, revised this paper critically, and gave final approval for the version to be submitted. ZL, HS, JD, XX, and JS contributed to data acquisition. ZL was responsible for analyzing the WES results, Sanger sequencing, zebrafish related experiment, and drafted the manuscript. XX performed part of the zebrafish experiment and mice breeding. HS collected the patients’ information. JS took part in WES analyzing. JD contributed to ITPR1’s expression chasing and revising this paper. All authors contributed to the article and approved the submitted version.

## Conflict of Interest

The authors declare that the research was conducted in the absence of any commercial or financial relationships that could be construed as a potential conflict of interest.

## Footnotes

1 https://www.broadinstitute.org/gatk/guide/best-practices

2 http://www.gene-tools.com/

3 http://sift.jcvi.org

4 http://genetics.bwh.harvard.edu/pph2/

## Supplementary Material

Supplementary Figure 1 **(A)** Diagram of morpholine design showing the zebrafish gene itpr1b and morpholino antisense strategies to block the ATG (ATG-MO) and the splice acceptor site of exon4 (e4i4 MO). **(B)** Effectiveness of itpr1b knockdown (itpr1b-e4i4-MO) was confirmed by RT-PCR at 120 hpf, ef1α used as the internal control.
