# Supplementary figures and images for: ITPR1 Mutation Contributes to Hemifacial Microsomia Spectrum
Source: Front Genet. 2021 Mar 4;12:616329. doi: 10.3389/fgene.2021.616329 (PMC7971309; doi:10.3389/fgene.2021.616329)

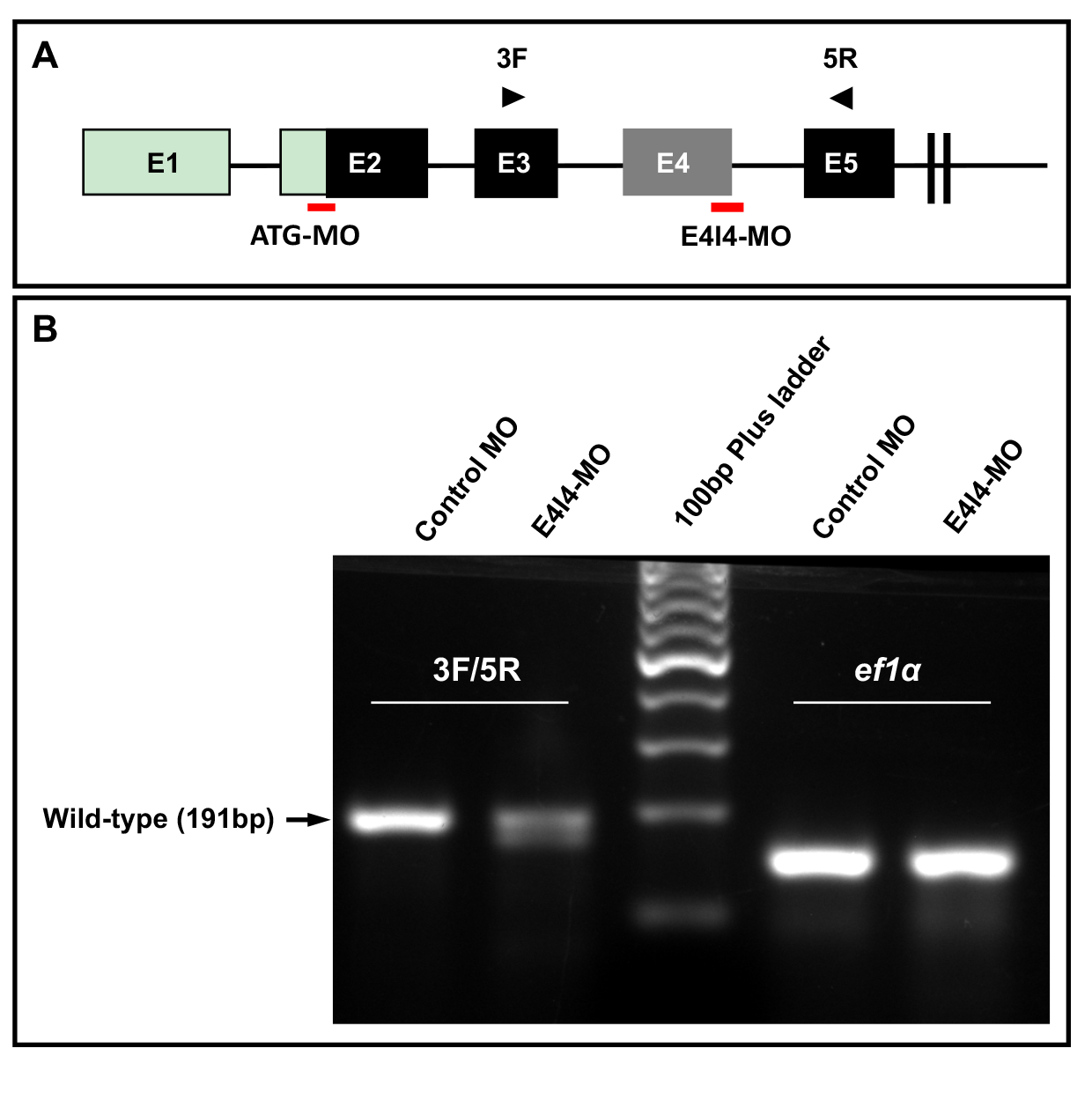

Supplement: Supplementary Figure 1 — (A) Diagram of morpholine design showing the zebrafish gene itpr1b and morpholino antisense strategies to block the ATG (ATG-MO) and the splice acceptor site of exon4 (e4i4 MO). (B) Effectiveness of itpr1b knockdown (itpr1b-e4i4-MO) was confirmed by RT-PCR at 120 hpf, ef1α used as the internal control. [file Image_1.tif]
